# Claims in orthopedic foot/ankle surgery, how can they help to improve quality of care? A retrospective claim analysis

**DOI:** 10.1007/s00590-020-02745-9

**Published:** 2020-07-26

**Authors:** Fay R. K. Sanders, Patricia Wimmer-Boelhouwers, Onno X. Dijt, Gino M. M. J. Kerkhoffs, Tim Schepers

**Affiliations:** 1 Trauma Unit, Amsterdam Movement Sciences, Amsterdam UMC, University of Amsterdam, Meibergdreef 9, 1105AZ Amsterdam, The Netherlands; 2 Trauma Unit, Amsterdam UMC, University of Amsterdam, Meibergdreef 9, 1105AZ Amsterdam, The Netherlands; 3 MediRisk, Van Deventerlaan 20, 3503 RK Utrecht, The Netherlands; 4 Orthopedic Surgery Department, Amsterdam UMC, University of Amsterdam, Meibergdreef 9, 1105AZ Amsterdam, The Netherlands

**Keywords:** Malpractice claims, Foot, Ankle, Orthopedic surgery, Trauma surgery

## Abstract

**Background:**

Orthopedic foot/ankle surgery is a high risk specialty when it comes to malpractice claims. This study aims to evaluate the incidence, characteristics and outcome of claims in this area.

**Methods:**

This was a retrospective, 10-year claim analysis, with data from an anonymous database. Baseline claim/claimant characteristics were collected from all orthopedic foot/ankle-related cases.

**Results:**

Of 460 claims in total, most were related to delay in/wrong diagnosis or to (complications of) elective surgical procedures. Whether a claim was settled was related to type of injury (fracture) and type of claim (diagnostic mistake). Median amount disbursed in settled claims was €12,549. Claim incidence did not increase over the years.

**Conclusion:**

Missed fracture diagnosis and “failed”/disappointing results of elective surgical procedures were the most common causes for claims. Sufficient knowledge of missed (foot) fractures and clear communication/expectation management before elective procedures could help to improve quality of healthcare and patient satisfaction.

## Introduction

Recently, the fear of receiving a malpractice claim has increased and the consequences of that fear, such as overtreatment or depression/burnout among medical professionals, are not insignificant [1, 2]. The number of claims and the life time chance of receiving a claim may have increased over the years [3]. Orthopedic surgery has been defined as a high-risk specialty when it comes to the probability of receiving a claim [4, 5]. Within the orthopedic field, claims concerning the lower extremity seem to result in larger settlements then the upper extremity [6, 7]. Moreover, the foot and ankle region accounts for a large number of claims [8–10]. However, research on causality and risk factors for claims in this area is limited. Most studies focusing on claims in orthopedic surgery have investigated spinal surgery, or elective procedures such as joint replacements [11–14].

Identifying the most common reasons for claims in a specific field can lead to healthcare improvements to avoid patient damages and claims in the future. In the 90′s, MediRisk, one of the 2 medical liability insurance companies in the Netherlands, analyzed national claim data and found that most claims in the emergency department were related to misdiagnosis or treatment of fractures and tendon injuries [15]. Following these results, new guidelines and a safety net were installed, comprising, among other things, a daily radiology meeting where every X-ray of the day was discussed with both radiologist and supervisor. A study on hand and wrist injury-related malpractice claims evaluated the effect of that intervention and identified a mild decline in missed fractures [16].

This study aims to evaluate: 1. the incidence of claims related to orthopedic foot/ankle surgery, 2. the most common characteristics of claimants and claims, and 3. consequences and outcome of claims, in order to identify opportunities to improve care of foot/ankle conditions.

## Methods

This is a retrospective, observational database study, investigating all claims related to foot/ankle injury within the scope of 10 years.

### Key aspects of medical liability in the Netherlands

In the Netherlands, patients file a claim for compensation against the hospital and not directed at the physician personally, as is common in some other countries. MediRisk, as a medical liability insurer for its member hospitals, handles the claims of these patients on behalf of the hospitals. The burden of proof for medical negligence, damages and causation lies with the patient. When medical negligence has been established, MediRisk will compensate all reasonably damages caused by the negligence. At present over 95% of all cases are settled outside of court.

### Data collection

The data for this study were collected from the anonymous database of Medirisk. Medirisk is one of the 2 medical liability insurance companies in the Netherlands and represents around 50% of all non-academic hospitals in the country. The database is roughly classified for (among other things) concerned body part, cause, treating specialty, care process and consequences of claims, but also contains short descriptions of the individual claims (often in layman’s terms). All claims filed between 1–1–2007 and 12–31–2018 classified as concerning the “foot”, “ankle”, “toes” or “lower leg”, with the treating specialty being “surgery”, “orthopedic surgery”, “radiology” or “emergency medicine” were extracted. In addition to this, all claims with a description containing the words foot, ankle, toes or lower leg (and all medical and layman’s synonyms for those words) in combination with all terms suggesting injury or surgery were acquired from the database (full search in Appendix). Claims which were filed after 2016 (due to proportion of claims still open), not concerning the foot or ankle, not concerning orthopedic/trauma surgery and claims concerning patients younger than 18 years old were excluded.

### Outcome measures

Extracted variables were (1) baseline characteristics such as age, gender and type of injury/affliction of the claimant, (2) treatment characteristics such as treating physician and operative treatment and (3) claim characteristics such as the type of claim (Table 1), consequences of the claim (both reported by claimant and medical expert), how the claim was handled (settled, declined, amicable settlement, closed without conclusion, negligible without harm) and what the costs were. These were displayed using descriptive statistics, reporting continuous variables in mean and standard deviation when normally distributed, in median and interquartile range when not normally distributed and categorical variables in numbers and percentages. Variables were compared between in- and excluded cases and between settled and declined claims using a Mann–Whitney *U* test for the continuous outcome measures and a Chi-Square for categorical outcome measures. Claim incidence was calculated per year corrected for the number of insured hospitals each year by separately depicting the constant group of hospitals (*n* = 42) and the number of hospitals that varied (*n* = 13).

**Table 1:** Classification in types of claims

Type of claim	Definition
Diagnosis	All claims concerning a missed, delayed or wrong diagnosis
Communication	All claims concerning a lack of or the wrong information in communication with the patient
Treatment	All claims concerning inadequate treatment (excluding treatment through surgery or medicaments)
Surgery	All claims concerning the indication, technical aspects or results of a surgical treatment
Medication	All claims concerning withholding of or prescription of the wrong medication
Care	All claims concerning problems in the hospital, located on the ward and not related to medication

### Study population

Out of the 909 claims that were extracted from the database, 449 were excluded because they were filed after 2016 (*n* = 78), not concerning the foot or ankle (*n* = 305), not regarding a orthopedic/trauma-surgical problem (*n* = 15), the claimant was under 18 y/o (*n* = 43), or because there was not enough information to classify the claim properly (*n* = 8), leaving 460 for analysis. Excluded cases did not differ significantly from the included claims in age (47.1, SD 19.6 vs. 46.5 SD 15.9) or gender (218, 48.6% vs. 198, 43.0% males) of the claimant. There was a difference in location of the injury, which was significantly more in the lower leg for excluded cases, (193 vs 0, *p* < 0.01). The treating specialty was more often a general surgeon in excluded cases (246, 54.8% vs. 196, 42.6%) and excluded cases were more likely to be surgery related (242, 53.9% vs. 200, 43.5%) whereas included cases were more likely related to diagnosis. The status of excluded claims was more often still undecided (69, 15.4% vs. 23, 5.0%).

## Results

### Claim incidence

**Fig. 1:**
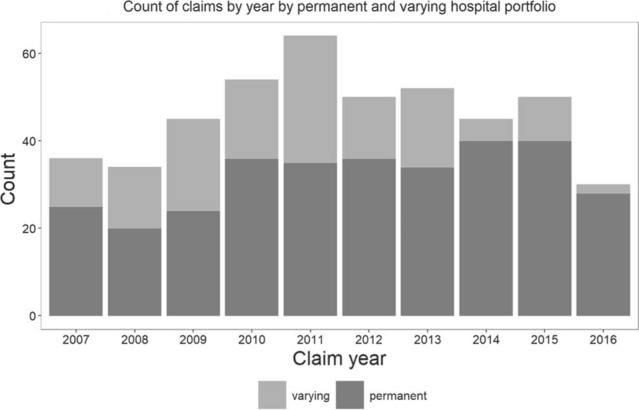
Claim incidence per year. This figure shows the total included number of filed claims per year. To correct for the number of insured hospitals which varied over the years, results are separately shown for the group of hospitals that was insured at MediRisk during the entire study duration (permanent) and for the group of hospitals that varied (varying)

The overall number of claims varied slightly per year, with the largest number of settled claims in 2014. Regarding only the constant group of insured hospitals, the peak in claim incidence was in 2014/2015 with a subsequent decline in 2016 (Fig. 1).

### Claim and claimant characteristics

**Fig. 2:**
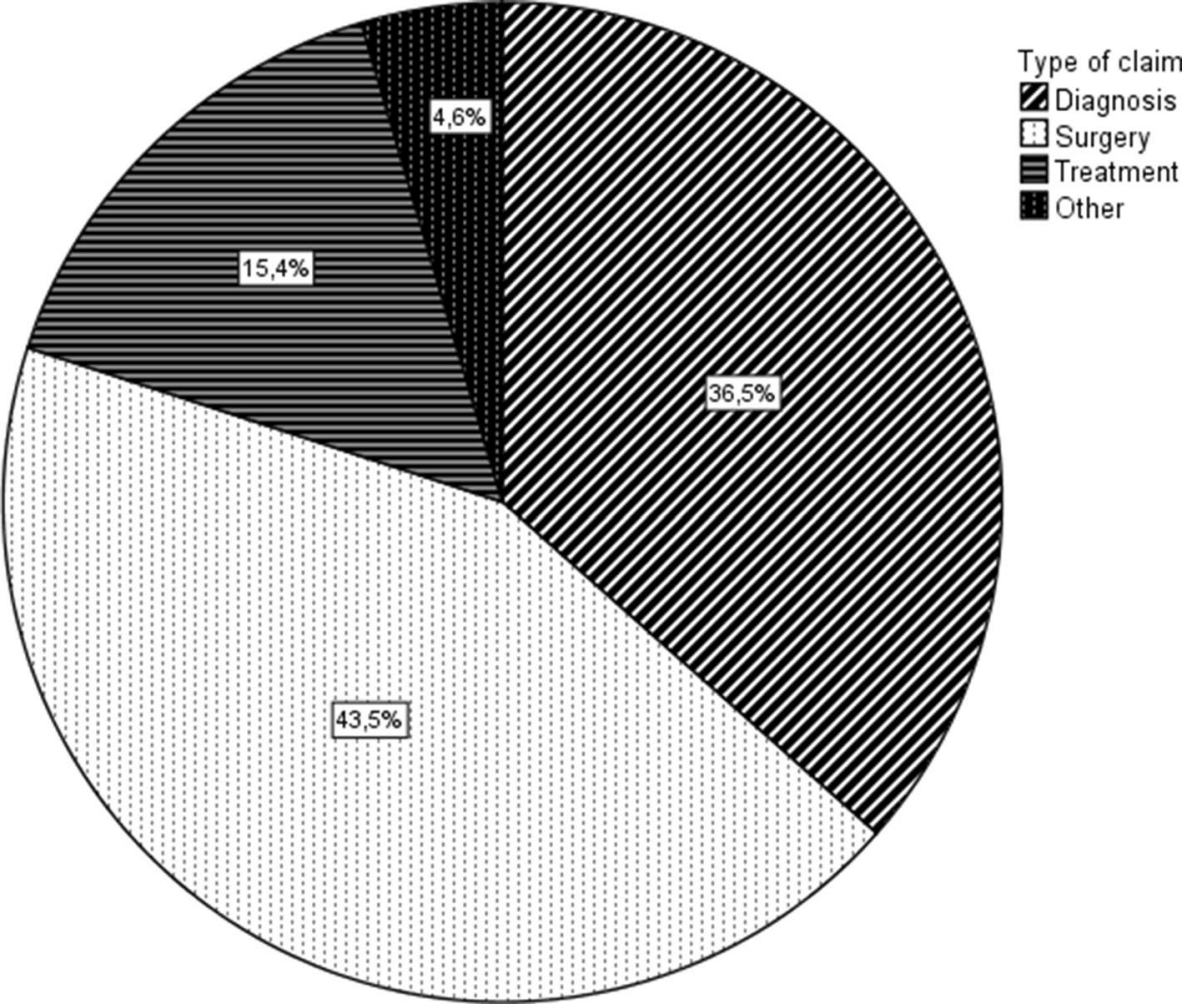
Type of claim distribution. This figure visually displays the distribution of types of claims as classified in Table 1

Baseline characteristics of the claimant and the treatment he/she received are shown in Table 2. The claimant was 46.5 years old on average and female in 56.7%. The injury/affliction that was most frequently underlying the claim was a fracture and 80% of claims were related to either (the outcome/complications of) an operative procedure, or to a missed/wrong diagnosis (Fig. 2).

**Table 2:** Baseline and surgical characteristics of claimants

Characteristics	*N* = 460
Age, mean, (SD)^a^	46.5 (15.9)
Gender, No of females (%)^b^	261 (56.7%)
Treating specialty	
Orthopedic surgeon	231 (50.2%)
(trauma) surgeon	196 (42.6%)
Radiologist	25 (5.2%)
ER	8 (1.7%)
Location of injury/affliction	
Ankle (%)	189 (41.1%)
Foot (%)	192 (41.7%)
Toes (%)	79 (17.2%)
Type of injury/affliction:	
Fracture	208 (45.2%)
Toe deformities^c^	66 (14.3%)
Tendon/capsular damage	38 (8.3%)
Infection	22 (4.5%)
Luxation	4 (0.9%)
CRPS	4 (0.9%)
Other^d^	53 (11.5%)
Unknown^e^	65 (14.1%)
Type of surgery (*N* = 200)	
Acute	48 (24%)
Elective	112 (56%)
Unclear^e^	40 (20%)

*N* number of patients, *SD* standard deviation, *ER* emergency room, *CRPS* complex regional pain syndrome

^a^In years, at time of the inflicted damage

^b^1 missing value

^c^E.g. hallux valgus, hammer/claw toe, ingrown toenail

^d^E.g. removal of foreign body, lipoma, neuroma, compartment syndrome, and other orthopedic pathology

^e^Unspecified underlying injury or procedure (e.g. complications after “foot surgery”)

A subgroup analysis of only fracture-related claims showed that these were mostly fractures of the ankle (111, 53.4%) or the foot (85, 40.9%) and the type of claim was diagnostic in 106 cases, 51.0%. Surgical claims and treatment-related claims represented 23.6% (*n* = 49) and 22.1% (*n* = 46), respectively, in fractures. A subgroup analysis of all surgical claims showed that these were related to an elective procedure in 112 (56.0%) of cases. The underlying injury/affliction was missing in 49 (24.5%), but of the remaining cases it was a fracture in 49 (32.5%) and a hallux valgus in 46 (30.4%) cases. A larger proportion of female claimants (128, 64%) was seen in this subgroup compared to the entire study population. When specifically looking at diagnostic claims, these concerned fractures in 106 (63.1%), followed by tendon injuries in 22 (13.1%). The location of the injury was the foot in 86 (51.2%), the ankle in 64 (38.1%) and the toes in 18 (10.7%). Age and gender did not differ from the rest of the study population.

### Claim outcome and consequences

Out of 460 claims, 142 (30.9%) were settled/plaintiff verdict, 259 (56.3%) was declined/defense verdict and of the remainder; 36 (7.8%) came to an amicable settlement, 21 (4.6%) were closed without a conclusion and 2 (0.4%) were negligent without causing the claimant harm. Differences in baseline, surgical and claim characteristics between settled claims and declined claims are shown in Table 3. The median amount in euros disbursed in settled claims was €12,549 with a maximum of €322,149, IQR: [€4264–€36,145]. When analyzing the data per year, the year 2011 has the highest median disbursed amount (€23,566, IQR: €6,969–€69,689). Amicable settlements were worth €2641 on average (IQR: €1391–€7275). The most expensive claim of €325,000 concerned a 35 y/o male with a wrongly placed ankle prosthesis, which resulted in revision surgery and ultimately an amputation because of continuous pain. The second most expensive claim (€225,000) concerned a 45 y/o female with an intra-articular ankle fracture who did not receive a CT/MRI in the emergency department and initially received a cast instead of operative treatment, causing a delay in proper treatment and unresolved pain/limited function. The third most expensive claim (€190,000) concerned a Weber C ankle fracture in a 45 y/o male with limited functional outcome due to an operative procedure not performed according to protocol (ages and awarded amounts were rounded to guard privacy of claimant).

**Table 3:** Baseline claim/claimant characteristics of settled and declined claims

Characteristics	Settled claims (*N* = 142)	Declined claims (*N* = 259)	*p*-value*
Age, mean, (SD)^a^	47.3 [35.4–58.6]	46.8 [32.9–58.6]	0.420
*Gender*^b^			0.636
Nr of males (%)	60 (42.3%)	117 (45.2%)	
Nr of females (%)	82 (57.7%)	141 (54.4%)	
*Treating specialty*			0.596
Orthopedic surgeon	68 (47.9%)	135 (52.1%)	
(trauma) surgeon	61 (43.0%)	109 (42.1%)	
Radiologist	9 (6.3%)	11 (4.2%)	
ER	4 (2.8%)	4 (1.5%)	
*Location of injury/affliction*			0.201
Ankle (%)	64 (45.1%)	101 (39.0%)	
Foot (%)	60 (42.3%)	108 (41.7%)	
Toes (%)	18 (12.7%)	50 (19.3%)	
*Type of injury/affliction*			0.024
Fracture	79 (55.6%)	103 (39.8%)	
Toe deformities^c^	13 (9.2%)	45 (17.4%)	
Tendon/capsular damage	11 (7.7%)	22 (8.5%)	
Infection	1 (0.7%)	18 (6.9%)	
Luxation	1 (0.7%)	2 (0.8%)	
CRPS	1 (0.7%)	3 (1.2%)	
Other^d^	13 (9.2%)	32 (12.4%)	
Unknown^e^	23 (16.2%)	34 (13.1%)	
*Type of surgery *(*N* = 200)			0.449
Acute	20 (29.4%)	24 (21.4%)	
Elective	34 (50.0%)	65 (58.0%)	
Unclear	14 (20.6%)	23 (20.5%)	
*Type of claim*			0.033
Surgery	68 (47.9%)	112 (43.2%)	
Diagnosis	58 (40.8%)	85 (32.8%)	
Treatment	13 (9.2%)	46 (17.8%)	
Medication	3 (2.1%)	12 (4.6%)	
Communication	0	4 (1.5%)	
Care	0	0	

*N* number of patients, *SD* standard deviation, *ER* emergency room, *CRPS* complex regional pain syndrome

*determined by Mann–Whitney *U* for age and with a Chi Square test for all other, categorical variables

^a^In years, at time of the inflicted damage

^b^1 missing value in “declined claims”

^c^E.g. hallux valgus, hammer/claw toe, ingrown toenail

^d^E.g. removal of foreign body, lipoma, neuroma, compartment syndrome, and other orthopedic pathology

^e^Unspecified underlying injury (e.g. complications after “foot surgery”)

As shown in Table 4, the 3 most common patient-reported consequences were functional restriction, pain and revision surgery or readmission. When looking at the objective consequences (confirmed by a medical expert), the most common one is delay (in either treatment or diagnosis).

**Table 4:** Claim consequences as reported by claimant and verified by medical expert

Consequence	Claimant	Objective
Functional restriction	94 (20.4%)	19 (4.1%)
Pain	88 (19.1%)	13 (2.8%)
Revision or readmission	74 (16.1%)	29 (6.3%)
Delay	70 (15.2%)	39 (8.5%)
Disappointing results	15 (3.3%)	3 (0.7%)

Consequences of claims as reported by the claimant and the objective consequences as determined by the medical expert working on the case. Results are displayed as number and percentage of all included claims (*N* = 460)

## Discussion

### Background and rationale

Events leading to filing a claim have a large impact on both the patient and the medical professionals involved [1]. Leaving patients unsatisfied goes directly against the fundamental motives of healthcare professionals to care for and cure patients. However, it is therefore especially important to evaluate the result of these good intentions. Analyzing claims gives us a unique chance to get a patient’s perspective on the quality of healthcare. Something that is particularly useful in a high-risk specialty such as foot/ankle surgery, with a large variety of pathology/injuries and a high rate of postoperative complications [17, 18]. This retrospective study therefore aimed to use foot/ankle surgery-related claims to identify opportunities to improve care of foot/ankle conditions.

### Claim incidence

The claim incidence did not seem to increase over the years. Although it is hard to draw conclusions because of the relatively small amount of claims per year in this subgroup, this seems comparable to literature. Klemann et al. have previously shown an increase in number of claims until 2012 and a stabilization after that in an investigation of all claims in the Netherlands from 2007 to 2016 [19]. A series of other studies could also not demonstrate an increase in the number of claims per year [4, 9]. However, there are also a few European studies that did find an increase in claim incidence [3, 7].

### Claimant characteristics

Considering the baseline characteristics of claimants, we found that claimants were on average young (46.5 y/o), female patients with a fracture. It has been previously reported that in general, elderly patients were less likely to file a claim [20]. In orthopedic surgery, this is supported by a Finnish study, linking all total hip and total knee replacements to the number of claims over a period of 5 years. They found that males and elderly patients were significantly less likely to file a claim, whereas increased comorbidity was positively associated with a claim [21]. However, a majority of male plaintiffs has also been described in a study looking at litigation after traumatic fractures [22]. Although we had no information about comorbidities, the claims in our cohort were mostly filed by young, female patients.

### Claim characteristics

As for the reasons of filing a claim, in this cohort the claim was often related to a delay in/wrong diagnosis or to (complications of) an elective operative procedure. Both in general and in orthopedic surgery, multiple authors have previously suggested diagnostic mistakes as the most common cause for a claim [9, 23]. Injuries of the foot often have a large impact on functional recovery and, within the extremities, the highest number of missed fractures [24, 25], which could also explain the high percentage of foot injuries in our cohort, especially in the subgroup analyses of diagnostic claims. Even though ankle injuries are much more common, the percentage of foot and ankle claims in our cohort was comparable. Many studies have stressed the large impact of foot injuries on functional outcome and quality of life [26]. The chance of filing a claim also seems to correlate to the patient reported outcome, as illustrated by Thornes et al. who found that patients pursuing compensation had significantly worse outcome scores, unrelated to objective outcome measures and whether they were operatively or conservatively treated for their calcaneal fracture [27]. Calder et al. confirmed this in their cohort of Lisfranc injuries and also identified delay in diagnosis and treatment as important independent predicting factors in filing a claim [28].

The other major reason for filing a claim in this cohort, surgical errors or complaints about the outcome, have also been previously described [10, 29]. Casali et al. also found that claims were most often related to a surgery and that the majority of those procedures were elective [9]. Other authors have mentioned a misbalance in expectations before surgery and the result that follows [10, 30]. This could very well explain the fact that most surgical claims are related to an elective procedure, since in acute setting expectations might be lower [31].

### Claim outcome and consequences

Regarding the claim outcome, previous studies from the Netherlands have reported a settlement rate of 8–25% of claims [32–34]. This number was higher in our study (30.9%). An explanation might be that, in recent years, claims are more often filed by legal representatives. It is possible that through a selection process by these representatives, claims are only filed when there is a high probability of a settlement. This is supported by the mildly decreasing rate of declined claims over the past 10 years as Klemann et al. have shown in an investigation of all claims in the Netherlands from 2007 to 2016 [19].

In this cohort, settled claims were more often related to a fracture and to a missed/wrong diagnosis than declined claims. Bjørslev et al. previously analyzed claims after operative treatment of ankle fractures to identify iatrogenic risk factors for a settled/compensated claim. In contrast to our study, they found that most settled claims concerned a problem in the perioperative phase, most often incongruity of the ankle joint. However, they had a small cohort of 51 claims and did not analyze declined claims or foot injuries [35]. The average amount of €12,549 that was disbursed to patients in a plaintiff verdict was comparable to that of other studies from the Netherlands [19, 33, 34]. The patient-reported consequences (functional restriction, pain and revision surgery or readmission) are compatible with the earlier mentioned theory of a misbalance in expectations of surgery and the result that follows [10, 30]. One can theorize that claims may have been prevented by sufficient expectation management and informing patient of the risks, especially since the common objective consequences are not the same as the patients reported ones.

### Limitations

This study is subjected to a few limitations. First of all, the information supplied to the medical liability insurance company is from a patient perspective. Although it is checked and rated by medical professionals, the information extracted from the database is primarily “patient-reported”, making it subjective. Specific diagnoses were often not reported. Another possible limitation from this patient’s perspective is that it is not possible to directly link the highest awarded amounts to the biggest “mistakes”. Often, the awarded amount also depends on the consequences and circumstances of an individual claimant. However, to improve healthcare by identifying bottlenecks, the patient’s perspective on hospital procedures might even be more valuable than the objective events. Therefore, the patient’s perspective is at the same time a major strength of this study, since patient’s satisfaction plays a big part in quality of care.

The second limitation is that, because of the patient-reported data, this study could not give a specialist perspective. It might have been valuable to identify how many claims a certain medical professional receives and what the risk factors for receiving a claim are. However, this is out of scope of the current trial and has been previously studied [9].

Finally, although it is important to compare results of this study with those of others, not all aspects can be compared between countries. Because of widely varying juridical claim procedures, information such as the rate of settled/declined claims and awarded amounts for settled claims, cannot be compared. Therefore, we were limited to a small number of previous cohorts from the Netherlands for these comparisons.

### Conclusion

Despite the limitations, we feel that this study provides valuable information for the orthopedic/ trauma surgical community. Although claims have been previously studied in orthopedic fracture surgery, this study represents the largest cohort that focuses on foot/ankle surgery [22, 35]. Given the high complication rate in this area of orthopedic surgery, it is important to identify pitfalls in (operative) care and communication, not only to prevent malpractice claims, but to improve the quality of care. In conclusion, the results of this study show that missed (foot) fractures and “failed”/disappointing results of elective operative procedures are the most common causes for claims. Although not all (alleged) medical errors can be avoided, some lessons can be learned from this conclusion. First of all, a high index of suspicion and adequate knowledge of the most commonly missed foot fractures could decrease the number of missed/wrong diagnoses in the acute setting and benefit timely and adequate treatment. Secondly, the importance of clear communication/expectation management before elective procedures is once more underlined.

## Data Availability

The anonymous database is available upon request.

## Code availability

N/A.

## References

[1] Balch CM, Oreskovich MR, Dyrbye LN, Colaiano JM, Satele DV, Sloan JA (2011). Personal consequences of malpractice lawsuits on American surgeons. J Am Coll Surg.

[2] Lyu H, Xu T, Brotman D, Mayer-Blackwell B, Cooper M, Daniel M (2017). Overtreatment in the United States. PLoS ONE.

[3] Buzzacchi L, Scellato G, Ughetto E (2016). Frequency of medical malpractice claims: the effects of volumes and specialties. Soc Sci Med.

[4] Jena AB, Seabury S, Lakdawalla D, Chandra A (2011). Malpractice risk according to physician specialty. N Engl J Med.

[5] Gómez-Durán EL, Martin-Fumadó C, Benet-Travé J, Arimany-Manso J (2018). Malpractice risk at the physician level: claim-prone physicians. J Forensic Leg Med.

[6] Matsen FA, Stephens L, Jette JL, Warme WJ, Posner KL (2013). Lessons regarding the safety of orthopaedic patient care: an analysis of four hundred and sixty-four closed malpractice claims. J Bone Jt Surg Ser A.

[7] Agout C, Rosset P, Druon J, Brilhault J, Favard L (2018). Epidemiology of malpractice claims in the orthopedic and trauma surgery department of a French teaching hospital: a 10-year retrospective study. Orthop Traumatol Surg Res.

[8] Fountain S, Brooks D, Butler D et al (2000) Managing orthopaedic malpractice risk: committee on professional liability. Rosemont Am Acad Orthop Surg 3–78

[9] Casali MB, Blandino A, Del Sordo S, Vignali G, Novello S, Travaini G (2018). Alleged malpractice in orthopaedics. Analysis of a series of medmal insurance claims. J Orthop Traumatol.

[10] Tarantino U, Giai Via A, Macrì E, Eramo A, Marino V, Marsella LT (2013). Professional liability in orthopaedics and traumatology in Italy. Clin Orthop Relat Res.

[11] Erivan R, Chaput T, Villatte G, Ollivier M, Descamps S, Boisgard S (2018). Ten-year epidemiological study in an orthopaedic and trauma surgery centre: are there risks involved in increasing scheduled arthroplasty volume without increasing resources?. Orthop Traumatol Surg Res.

[12] Machin JT, Hardman J, Harrison W, Briggs TWR, Hutton M (2018). Can spinal surgery in England be saved from litigation: a review of 978 clinical negligence claims against the NHS. Eur Spine J.

[13] Agarwal N, Gupta R, Agarwal P, Matthew P, Wolferz R, Shah A (2018). Descriptive analysis of state and federal spine surgery malpractice litigation in the United States. Spine Phila Pa 1976.

[14] Patterson DC, Grelsamer R, Bronson MJ, Moucha CS (2017). Lawsuits after primary and revision total knee arthroplasty: a malpractice claims analysis. J Am Acad Orthop Surg.

[15] MediRisk (2019) Schade op de Spoedeisende Hulp n.d. https://www.medirisk.nl/kennisbank-preventie/hoge-risicos/schade-op-de-spoedeisende-hulp. Accessed December 5, 2019

[16] Zegel M, Selles RW, van der Heijden EPA, Zuidam JM (2017) Nog steeds onvoldoende kennis over hand- en polsletsels op de SEH in Nederland. Med Contact (Bussum)

[17] Feilmeier M, Dayton P, Sedberry S, Reimer RA (2014). Incidence of Surgical site infection in the foot and ankle with early exposure and showering of surgical sites: a prospective observation. J Foot Ankle Surg.

[18] Wiewiorski M, Barg A, Hoerterer H, Voellmy T, Henninger HB, Valderrabano V (2015). Risk factors for wound complications in patients after elective orthopedic foot and ankle surgery. Foot Ankle Int.

[19] Klemann DMTV, Mertens HJMM, Van Merode GG (2018). Meer en hogere schadeclaims analyse van schadec laims in de Neder landse ziekenh uis zorg van 2007–2016. Ned Tijdschr Geneeskd.

[20] Bismark MM, Brennan TA, Davis PB, Studdert DM (2006). Claiming behaviour in a no-fault system of medical injury: a descriptive analysis of claimants and non-claimants. Med J Aust.

[21] Järvelin J, Häkkinen U, Rosenqvist G, Remes V (2012). Factors predisposing to claims and compensations for patient injuries following total hip and knee arthroplasty. Acta Orthop.

[22] Ahmed SA, Defroda SF, Naqvi SJ, Eltorai AEM, Hartnett D, Ruddell JH (2019). Orthopaedic malpractice litigation following traumatic fracture. J Bone Joint Surg Am.

[23] Saber Tehrani AS, Lee HW, Mathews SC, Shore A, Makary MA, Pronovost PJ (2013). 25-Year summary of US malpractice claims for diagnostic errors 1986–2010: an analysis from the National Practitioner Data Bank. BMJ Qual Saf.

[24] Wei CJ, Tsai WC, Tiu CM, Wu HT, Chiou HJ, Chang CY (2006). Systematic analysis of missed extremity fractures in emergency radiology. Acta Radiol.

[25] Hallas P, Ellingsen T (2006). Errors in fracture diagnoses in the emergency department – characteristics of patients and diurnal variation. BMC Emerg Med.

[26] Schepers T, Rammelt S (2017). Complex foot injury: early and definite management. Foot Ankle Clin.

[27] Thornes BS, Collins AL, Timlin M, Corrigan J (2002). Outcome of calcaneal fractures treated operatively and non-operatively. The effect of litigation on outcomes. Ir J Med Sci.

[28] Calder JDF, Whitehouse SL, Saxby TS (2004). Results of isolated Lisfranc injuries and the effect of compensation claims. J Bone Jt Surg Ser B.

[29] Rynecki ND, Coban D, Gantz O, Gupta R, Ayyaswami V, Prabhu AV (2018). Medical malpractice in orthopedic surgery: a westlaw-based demographic analysis. Orthopedics.

[30] Morris JA, Carrillo Y, Jenkins JM, Smith PW, Bledsoe S, Pichert J (2003). Surgical adverse events, risk management, and malpractice outcome: morbidity and mortality review is not enough. Ann Surg.

[31] Stewart RM, Johnston J, Geoghegan K, Anthony T, Myers JG, Dent DL (2005). Trauma surgery malpractice risk: perception versus reality. Ann Surg.

[32] Delavary BM, Cremers JEL, Ritt MJPF (2010). Hand and wrist malpractice claims in the Netherlands: 1993–2008. J Hand Surg Eur.

[33] Elshove-Bolk J, Simons M, Cremers J, van Vugt A, Burg M (2004). A description of emergency department-related malpractice claims in the Netherlands: closed claims study 1993–2001. Eur J Emerg Med.

[34] Zengerink I, Reijman M, Mathijssen NMC, Eikens-Jansen MP, Bos PK (2016). Hip arthroplasty malpractice claims in the Netherlands: closed claim study 2000–2012. J Arthroplasty.

[35] Bjørslev N, Ebskov LB, Mersø C, Wong C (2018). Complications and patient-injury after ankle fracture surgery—A closed claim analysis with data from the Patient Compensation Association in Denmark. Injury.

